# Protocol for a randomised controlled trial to investigate the effect of home- and gym-based resistance exercise training on glycaemic control, body composition and muscle strength

**DOI:** 10.1186/s13063-020-04480-2

**Published:** 2020-06-22

**Authors:** Ebaa Al Ozairi, Dalal Alsaeed, Dennis Taliping, Mohamad Jalali, Abeer El Samad, Anant Mashankar, Etab Taghadom, Nicola Guess, Jason M. R. Gill, Naveed Sattar, Cindy Gray, Paul Welsh, Stuart R. Gray

**Affiliations:** 1 Medical Division, Dasman Diabetes Institute, P.O.Box 1180, Dasman, Kuwait; 2 Ministry of Health, Jamal Abdel Nasser Street, Sulaibkhat, 13001 Kuwait; 3 Institute of Cardiovascular and Medical Sciences, University of Glasgow, Glasgow, G12 8TA UK; 4 Institute of Health and Wellbeing, University of Glasgow, Glasgow, UK

**Keywords:** Diabetes, Resistance exercise, Unsupervised, Home-based, Glycaemic control

## Abstract

**Background:**

Resistance exercise is known to be effective in reducing glycated haemoglobin (HbA1c) in people with type 2 diabetes. However, studies, so far, have employed supervised resistance exercise in a laboratory or gym facility which limits the future translation of such exercise in to clinical practice and recommendations. Our primary aim, therefore, is to test the hypothesis, in a randomized controlled trial, that home-based resistance exercise training and gym-based resistance exercise training both reduce HbA1c levels in people with type 2 diabetes compared to control. We will also investigate the effects of home- and gym-based resistance exercise training on muscle strength and body composition.

**Methods:**

The current study is a three-arm randomised controlled trial which will be conducted with 150 eligible people with type 2 diabetes to compare home-and gym-based resistance exercise training with usual care in Kuwait. The interventions will be delivered by exercise specialists and last for 32 weeks. The primary outcomes are HbA1c with secondary outcomes measuring muscle function, body composition, physical activity and quality of life.

**Discussion:**

Ethical approval has been granted by the Dasman Diabetes Institute ethical review committee (RA/197/2019). Study findings will be disseminated through presentation at scientific conferences and in scientific journals.

**Trial registration:**

NCT04136730: Retrospectively registered on 21 October 2019

## Administrative information

Note: the numbers in curly brackets in this protocol refer to SPIRIT checklist item numbers. The order of the items has been modified to group similar items (see http://www.equator-network.org/reporting-guidelines/spirit-2013-statement-defining-standard-protocol-items-for-clinical-trials/).

**Table:** 

Title {1}	Protocol for a randomised controlled trial to investigate the effect of home and gym-based resistance exercise training on glycaemic control, body composition and muscle strength
Trial registration {2a and 2b}.	Retrospectively registered on 21 October 2019, trial registration number NCT04136730.
Protocol version {3}	Version 2. 4 September 2019.
Funding {4}	The study is funded by a grant from Monteral Medical International, Kuwait to University of Glasgow.
Author details {5a}	Ebaa Al Ozairi^1,2^, Dalal Alsaeed^1,3^, Dennis Taliping^1^, Mohamad Jalali^1,3^, Abeer El Samad^1^, Anant Mashankar^1^, Etab Taghadom^1,3^, Nicola Guess^1^, Jason MR Gill^5^, Naveed Sattar^5^, Cindy Gray^4^, Paul Welsh^5^, Stuart R Gray^5^ ^1^ Medical Division, Dasman Diabetes Institute, Kuwait ^2^ Department of Medicine, Faculty of Medicine, Kuwait University ^3^ Ministry of Health, Kuwait ^4^ Institute of Health and Wellbeing, University of Glasgow ^5^ Institute of Cardiovascular and Medical Sciences, University of Glasgow EAO, NG, JG, NS, PW and SRG were responsible for the conception of the work. EAZ and NS were responsible for clinical aspects of the study design. SRG, JG, NG and DT were responsible for responsible for the exercise aspects of the study design. NJ, DT and EAO were responsible for the recruitment plan, AES, SRG, EAO and ET were responsible for the design of the continuous glucose monitoring sub-study. AM, JG, EAO and SRG were responsible for the imaging aspects of the study design. PW was responsible for the statistical analysis plan for the study. SRG and EAO drafted the manuscript and all other authors revised it critically for important intellectual content and give final approval of the version to be published and agree to be accountable for all aspects of the work in ensuring that questions related to the accuracy or integrity of any part of the work are appropriately investigated and resolved.
Name and contact information for the trial sponsor {5b}	Ministry of Health, Kuwait
Role of sponsor {5c}	The sponsor had no role in the study design; collection, management, analysis, and interpretation of data; writing of the report; and the decision to submit the report for publication, and will have no ultimate authority over any of these activities.

## Introduction

### Background and rationale {6a}

The main role of muscle is to allow body movements via the generation of force, with the importance of this highlighted in conditions associated with muscle mass loss, such as sarcopenia [1]. Additionally, skeletal muscle has a critical, but often overlooked, role in metabolism [2]. For example, skeletal muscle is the primary protein store in the body, and during starvation [3] or conditions such as acquired immune deficiency syndrome (AIDS), can provide gluconeogenic precursors which are crucial for survival [4]. On top of this, as muscle is the primary site for glucose disposal in the body, it is therefore important in metabolic conditions such as diabetes [5]. Extending this further, the association of muscle in lifelong health is reflected by data demonstrating the association of muscle mass/function with mortality. This is highlighted by our recent work where we demonstrated, in ~ 500,000 participants (40–69 years) from the UK Biobank, that each 5 kg lower grip strength was associated with a higher risk of all-cause mortality [HR (95% CI) men 1.16 (1.15 to 1.17) and women 1.20 (1.17 to 1.23)] as well as a broad variety of other health outcomes [6]. Similarly, we have demonstrated that the increased risk of all-cause and CVD mortality in people with type 2 diabetes (T2D) is attenuated in those with high grip strength (CVD mortality with low [HR (95% CI) 4.05 (2.7 to 5.80)] vs high [HR 1.46 (0.87 to 2.46)], both relative to people without T2D with low grip strength) [7]. Taking this evidence together, this indicates that the maintenance of muscular mass and strength is of clear importance for public health.

Muscle strength is determined through a combination of multiple modifiable and non-modifiable factors. The primary modifiable factors are genetics with data indicating that hand grip strength is ~ 50% hereditable [8]. On the non-modifiable side, the primary factors important for the maintenance of muscle mass/strength are nutrition, particularly sufficient protein intake (e.g., [9]), and physical activity, particularly resistance exercise which is even effective in nonagenarians [10]. Resistance exercise has been shown, in a wide variety of populations, to be efficacious in increasing muscle mass and function, increasing basal metabolic rate, reducing blood pressure, improving blood lipids and glycaemic control [11]. Indeed, a meta-analysis in 2011 found that resistance exercise training can result in a 0.6% reduction in HbA1c in people with type 2 diabetes [12].

It is clear, therefore, that targeting increased muscle mass via resistance exercise may be effective in improving glycaemic control in people with type 2 diabetes, yet very few people participate in such exercise [13, 14]. One barrier to participation in resistance exercise is that access to equipment in specialised exercise facilities is required. This is a particular problem for resistance exercise, with aerobic exercise activities, such as walking/jogging, freely accessible to all without any training or expensive equipment. The majority of studies, which have demonstrated the efficacy of resistance exercise in reducing HbA1c, have been carried out in specialised exercise facilities/laboratories with each session supervised by a qualified instructor. There is the need, therefore, to develop a resistance exercise programme that is more accessible to the wider population. To date, little is known about the effectiveness or feasibility of home-based resistance training, without supervision, in people with type 2 diabetes.

### Objectives {7}

Our primary aim is to test the hypothesis, in a randomized controlled trial, that home-based resistance exercise training and gym-based resistance exercise training both reduce HbA1c levels in people with type 2 diabetes compared to control. We will also investigate the effects of home- and gym-based resistance exercise training on muscle strength and body composition.

### Trial design {8}

The current study is a three-arm randomised controlled trial comparing usual care plus home- or gym-based resistance exercise training interventions versus a usual care control group maintaining habitual physical activity levels. Eligible participants will be identified from clinics and via adverts places locally and on social media and we anticipate recruiting 150 participants with type 2 diabetes to the study. Baseline data will be measured prior to randomisation, and follow-up data will be collected 16 weeks and 32 weeks after randomisation adjusted for any delays in the start of the intervention post-randomisation.

## Methods: participants, interventions and outcomes

### Study setting {9}

This is a single-centre, DASMAN diabetes institute, three-arm randomised controlled trial.

### Eligibility criteria {10}

Potential participants will receive a mailed prestudy invite which will provide details on study information and details around eligibility. If still interested, potential participants will be invited to the research site where eligibility will be determined based on the inclusion and exclusion criteria (Table 1).

**Table 1:** Inclusion and exclusion criteria

In order to be considered eligible for participation in the study they must:	
Criterion	Characteristic of eligible participants
1	Be male or female aged ≥ 21 years at time of consent
2	Have physician diagnosed type 2 diabetes (HbA1c of 48 mmol/mol or over)
3	Have had no changes in anti-diabetic medication in the last 3 months
4	Have a BMI < 5 kg m^−2^
5	Have a blood pressure < 160/100 mmHg
6	Not be receiving insulin therapy
7	No currently, or in the last year, be participating in any vigorous aerobic activity (> 1 h per week)—defined a activity requiring a large amount of effort which causes rapid breathing and a substantial increase in heart rate
8	Not currently be participating in any resistance exercise training
9	Be a Kuwaiti resident
10	Not have any other condition that prevents exercising safely

### Who will take informed consent? {26a}

Written consent will be obtained by a member of the study team during a visit to the research site for the baseline assessment, with no study procedures taking place until consent has been obtained. At the baseline visit, a member of the study team will confirm eligibility.

### Additional consent provisions for collection and use of participant data and biological specimens {26b}

Not applicable

## Interventions

### Explanation for the choice of comparators {6b}

Following the baseline assessment, patients will be randomised to the home- or gym-based resistance exercise group or the control group (1:1:1 ratio) using a computer generated randomisation sequence. Randomisation will be stratified by sex.

### Intervention description {11a}

#### Control

Patients in the control group will receive usual clinical care and will be instructed to maintain their normal dietary and physical activity habits.

#### Home-based resistance exercise

Participants assigned to the home-based resistance exercise group will be asked to perform exercises 3 times a week for the intervention period. Each session will begin with a 5-min warm-up and end with a 5-min cool-down, walking on the spot/stretching. Exercises will target all major muscle groups. The exercises will be press-ups, band lateral raises, band seated low row, squat, lunge, calf raise, and plank. The order of each session will be (1) squat, (2) press up, (3) calf raise, (4) band seated low row, (5) lunge, (6) band lateral raise and (7) plank. Where participants are unable to perform any of the exercises, suitable alternatives will be sought.

The first 3 sessions of the exercise will be performed, within our exercise facility, under the supervision of a qualified exercise specialist to ensure that the participants are comfortable with the exercises and are performing them appropriately. During these sessions, participants will perform 5–10 repetitions of each exercise (tiring but comfortably achievable). A similar session, within the gym facility, will take place at week 4 and then every 4 weeks of the study to overcome any issues with the exercises and to ensure progression to an appropriate intensity. In weeks 2–4, participants will perform, during each session, a single set of each exercise with this set performed to voluntary muscular failure—defined as not being able to perform another repetition. In weeks 5–8, this will progress to 2 sets of each exercise with both sets performed to voluntary muscular failure, in each session. In weeks 9–32, 3 sets of each exercise will be performed with all sets to voluntary muscular failure, in each session. When multiple sets (weeks 5 onwards) of each exercise are performed, these will be sequentially with 2 min rest between each set.

#### Gym-based resistance exercise

Participants assigned to the gym-based resistance exercise group will undertake 3 supervised sessions per week. Each session will begin with a 5-min warm-up and end with a 5-min cool-down, walking or cycling and stretching. The exercises performed during each session will consist of leg press, calf press, leg curl, chest press, lateral raise, seated row, and plank. The order of the session will be (1) leg press, (2) chest press, (3) calf press, (4) seated row, (5) leg curl, (6) lateral raise and (7) plank. Where participants are unable to perform any of the exercises, suitable alternatives will be sought.

Exercises will be performed at 65–80% one repetition maximum (1RM), and this will be re-tested every 4 weeks of the intervention and the load adjusted accordingly. In week 1, participants will perform, during each session, a single set of 5–10 repetitions of each exercise (tiring but comfortably achievable) to ensure they are comfortable with the exercises and are performing these in the correct form. In weeks 2–4, participants will perform, during each session, a single set of each exercise with this set performed to voluntary muscular failure—defined as not being able to perform single another repetition. In weeks 5–8, this will progress to 2 sets of each exercise with both sets performed to voluntary muscular failure, in each session. In weeks 9–32, 3 sets of each exercise will be performed with all sets performed to voluntary muscular failure, in each session. When multiple sets (weeks 5 onwards) of each exercise are performed, these will be sequentially with 2 min rest between each set.

### Criteria for discontinuing or modifying allocated interventions {11b}

If patients are not able to carry out any of the exercise, then the trainers will work with the patient to develop a suitable alternative exercise.

### Strategies to improve adherence to interventions {11c}

For participants in the home-based group, the trainer will send a message to each participant on the day of their training session to remind them to carry out the exercise and to respond to say they have done it. An exercise log will also be provided for participants.

Those in the gym-based group will be sent a reminder message to ensure attendance at the supervised exercise sessions within our gym facility.

### Relevant concomitant care permitted or prohibited during the trial {11d}

Usual clinical care will be maintained for all patients during the trial.

### Provisions for post-trial care {30}

Usual clinical care will be maintained for all patients following completion of the trial.

### Outcomes {12}

Baseline characteristics will be collected—age, sex, blood pressure, duration of diabetes, current medications, current comorbidities and blood lipids.

All outcomes measures will be assessed at baseline, 16 weeks and 32 weeks in all participants.

#### Primary outcome measure

The primary outcome measure is change in HbA1c from baseline to 32 weeks.

#### Secondary outcome measures

Secondary outcome measures are achieved:

- DEXA (GE, Lunar body composition scan) derived whole body and regional lean mass.
- Functional abilities as assessed by the short performance physical battery test [15].
- Grip strength (measured 3 times in each hand using a Jamar Dynamometer)
- One repetition maximum for leg extension and lateral pull down.
- Number of push-ups (full or on knees) completed.
- DEXA derived fat mass and distribution.
- Physical activity levels measured by wrist-worn accelerometer (GENEActiv) for 7 days.
- Quality of life: EQ-5D-DL questionnaire.
- Current medications.
- Blood samples will also be stored for potential measurement of other biomarkers of interest.

#### Optional sub-study

Participants will also have the option to have liver fat measured by MRI and also 5-day continuous glucose monitoring.

#### Qualitative evaluation

A sub-group of participants will be invited for qualitative interviews at weeks 16 and 32 with an equal number of participants (*n* = 15) from each of the home- and gym-based resistance exercise groups. Participants will be invited to take part once they have left or completed the study and semi-structured interviews will take place at the Dasman Diabetes Institute (DDI) in person. All interviews will be audio-recorded and transcribed verbatim, anonymised during transcription and checked for accuracy.

Participants’ experiences and the acceptability of the interventions along with barriers and facilitators will be explored using principles of grounded theory.

### Participant timeline {13}

**Fig. 1:**
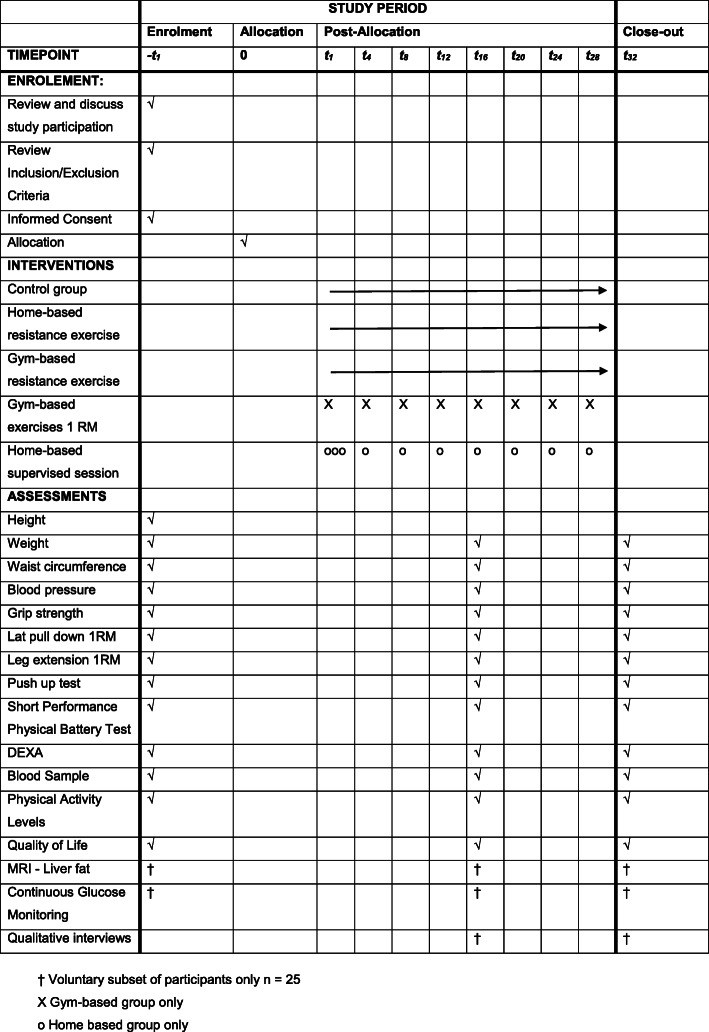
Schedule of Enrolement, Interventions and Assessments

The schedule of enrolement, interventions and assessments for participants is shown in Fig. 1.

### Sample size {14}

Previous research has shown that resistance exercise training can result in an ~ 0.6% decrease in HbA1c [12], with a 0.5% in HbA1c also being classified as a clinically relevant change. To detect a 0.5% decrease in HbA1c (standard deviation within groups 0.79%) in the home-based group or gym group versus control, we would require 40 participants per group at 80% power at the 5% significance level. We will recruit 50 to each group to account for drop out.

### Recruitment {15}

People with type 2 diabetes will be recruited:

- From DDI by usual care dieticians, physicians, nurses and trainers.
- Using advertisements via multiple media outlets that include texts, WhatsApp, flyers, social media and posters. These will target potential participants, primary health care centres and hospitals. Some advertisements will be designed by the public relations team at DDI.

## Assignment of interventions: allocation

### Sequence generation {16a}

Following the baseline assessment, patients will be randomised to the home- or gym-based resistance exercise group or the control group (1:1:1 ratio) using a computer-generated randomisation sequence. Randomisation will be stratified by sex.

### Concealment mechanism {16b}

Blinding of patients and research staff to the interventions is not possible but the staff and patients will be blinded to the sequence and all data will be analysed blind to allocation.

### Implementation {16c}

The allocation sequence will be generated within the electronic database (www.castoredc.com). Patients will be enrolled by a member of the research team.

## Assignment of interventions: blinding

### Who will be blinded {17a}

Blinding of patients and research staff to the interventions is not possible but all data will be analysed blind to allocation.

### Procedure for unblinding if needed {17b}

The current study design is open label with no blinding, and thus, unblinding will not occur.

## Data collection and management

### Plans for assessment and collection of outcomes {18a}

All outcome data will be collected according to developed standard operating procedures.

### Plans to promote participant retention and complete follow-up {18b}

No further strategies, than those described to maintain engagement with intervention, are planned.

### Data management {19}

All data will be kept in a secure storage area with access only to the study staff or on an online database (www.castoredc.com).

### Confidentiality {27}

Access to the collated participants’ data will be restricted to the principal investigator and appropriate research study staff as required. All laboratory samples, completed forms, reports and other records will be identified using a unique participant ID number to maintain participant confidentiality.

### Plans for collection, laboratory evaluation and storage of biological specimens for genetic or molecular analysis in this trial/future use {33}

Blood samples will be collected via venepuncture and analysed in our clinical biochemistry laboratories.

## Statistical methods

### Statistical methods for primary and secondary outcomes {20a}

All statistical analyses will be carried out according to a detailed Statistical Analysis Plan, to be finalised prior to database lock. We will use an intention to treat approach for analysis, using baseline observation carried forward for those who do not attend the 16 weeks visit, and last observation carried forward at the 32 week visit. This will estimate the effect of the interventions. The primary (and secondary) outcomes (all continuous) will be analysed in a hierarchical fashion, first analysing the change in outcome in the gym-based group versus control at 16 weeks at 5% level of significance; this is the group we hypothesise will have the greatest effect size. If this first difference is found to be significant, the home-based group will also be compared to control at the 5% level, and if this is significant, the gym-based group will be compared to the home-based group at the 5% level. The overall type I error will not exceed 5% for each outcome because of the hierarchical nature of the testing. Comparison of the randomised groups at 32 weeks will be conducted in a similar fashion, as a secondary analysis, at the 5% level. As a secondary analysis, we will conduct a per protocol ‘completer’s analyses’ of those who attended at least the 16-week visits (irrespective of their protocol adherence or other characteristics after randomisation). This is a less conservative estimate of the effect of the intervention. Finally, in the per protocol group, we will also investigate the association and correlation of change in HbA1C with change in secondary outcomes.

### Interim analyses {21b}

No interim analysis is planned in the current study.

### Methods for additional analyses (e.g., subgroup analyses) {20b}

No additional analyses are planned for the current study.

### Methods in analysis to handle protocol non-adherence and any statistical methods to handle missing data {20c}

Non-adherence will be handled through the use of intention to treat analysis in the primary analysis. Missing data will be handled by baseline observation carried forward in the primary analysis. Due to the randomised design, multivariable models will not be used to adjust for confounding, and therefore missing covariates will not impact the primary analysis.

### Plans to give access to the full protocol, participant level-data and statistical code {31c}

The data sets analysed and the codes used for these analyses are available from the corresponding author on reasonable request.

## Oversight and monitoring

### Composition of the coordinating Centre and trial steering committee {5d}

#### Principal investigator

- Design and conduct of trial
- Preparation of protocol and revision
- Preparation of study materials and case report forms
- Organise steering committee meetings
- Publication of study reports

#### Steering committee (see title page for members)

- Agreement of final protocol
- All lead investigators will be members
- Recruitment of patients and liasing with principal investigators
- Reviewing progress of study and if needed agreeing changes to protocol

#### Trial management committee (principal investigator, study coordinator, researchers)

- Study planning
- Adverse event reporting
- Responsible for trial master file
- Budget control
- Data verification

### Composition of the data monitoring committee, its role and reporting structure {21a}

Not applicable.

### Adverse event reporting and harms {22}

There are unlikely to be major safety issues with our proposed non-pharmacological interventions. However, if any safety concerns or incidents arise, we will follow our standard operating procedures for reporting adverse events in a non-Clinical Trial of an Investigational Medicinal Product (non-CTIMP) studies.

### Frequency and plans for auditing trial conduct {23}

Formal audits will occur every 6 months.

### Plans for communicating important protocol amendments to relevant parties (e.g. trial participants, ethical committees) {25}

Any amendments to the protocol will be approved by the local ethics committee prior to implementation. All investigators and patients enrolled in the trial will be informed.

## Dissemination plans {31a}

The study findings will be disseminated primarily via conference presentations and scientific papers.

## Discussion

The current trial is a single-centre, three-arm randomised controlled trail. It has been designed to test the effectiveness of home- and gym-based resistance exercise training over a period of 32 weeks in people with type 2 diabetes. It is known that resistance exercise training can effectively reduce HbA1c in people with type 2 diabetes [12] and the current study will answer whether delivery of such an intervention without regular supervision and outside of a gym-based facility is also effective, potentially allowing a more pragmatic delivery of resistance exercise within clinical practice.

## Trial status

The study began recruitment in August 2019 and will be ongoing until August 2020 (anticipated). Current protocol: version 2 (04.09.2019).

## Data Availability

Any data required to support the protocol can be supplied on request.
