# Correction to: Protocol for a randomised controlled trial to investigate the effect of home- and gym-based resistance exercise training on glycaemic control, body composition and muscle strength

**DOI:** 10.1186/s13063-020-04597-4

**Published:** 2020-07-15

**Authors:** Ebaa Al Ozairi, Dalal Alsaeed, Dennis Taliping, Mohamad Jalali, Abeer El Samad, Anant Mashankar, Etab Taghadom, Nicola Guess, Jason M. R. Gill, Naveed Sattar, Cindy Gray, Paul Welsh, Stuart R. Gray

**Affiliations:** 1 Medical Division, Dasman Diabetes Institute, P.O.Box 1180, Dasman, Kuwait; 2 Ministry of Health, Jamal Abdel Nasser Street, 13001 Sulaibkhat, Kuwait; 3 Institute of Cardiovascular and Medical Sciences, University of Glasgow, Glasgow, G12 8TA UK; 4 Institute of Health and Wellbeing, University of Glasgow, Glasgow, UK

**Correction to: Trials 21, 557 (2020)**

**https://doi.org/10.1186/s13063-020-04480-2**

Following publication of the original article [1], the authors notified us of a few changes to the study protocol that had to be made due to the COVID-19 pandemic lockdown which has resulted in the full closure of local gym facilities.

The gym based supervised resistance exercise group had to be, for this reason, left out of the study. This leaves the control and home-based exercise groups, which are able to continue during the pandemic.

The objectives were “to test the hypothesis, in a randomized controlled trial, that home-based resistance exercise training and gym-based resistance exercise training both reduce HbA1c levels in people with type 2 diabetes compared to control. We will also investigate the effects of home- and gym-based resistance exercise training on muscle strength and body composition.” These will now be changed as follows: “to test the hypothesis, in a randomized controlled trial, that home-based resistance exercise training reduces HbA1c levels in people with type 2 diabetes compared to control. We will also investigate the effects of home-based resistance exercise training on muscle strength and body composition.” We will adjust our statistical analysis plan accordingly to consider this change in study design and objectives.
